# Time Courses of Changes in Phospho- and Total- MAP Kinases in the Cochlea after Intense Noise Exposure

**DOI:** 10.1371/journal.pone.0058775

**Published:** 2013-03-06

**Authors:** Yukihide Maeda, Kunihiro Fukushima, Ryotaro Omichi, Shin Kariya, Kazunori Nishizaki

**Affiliations:** Department of Otolaryngology – Head and Neck Surgery, Okayama University Graduate School of Medicine, Dentistry and Pharmacy, Okayama, Japan; University of South Florida, United States of America

## Abstract

Mitogen-activated protein kinases (MAP kinases) are intracellular signaling kinases activated by phosphorylation in response to a variety of extracellular stimuli. Mammalian MAP kinase pathways are composed of three major pathways: MEK1 (mitogen-activated protein kinase kinase 1)/ERK 1/2 (extracellular signal-regulated kinases 1/2)/p90 RSK (p90 ribosomal S6 kinase), JNK (c-Jun amino (N)-terminal kinase)/c-Jun, and p38 MAPK pathways. These pathways coordinately mediate physiological processes such as cell survival, protein synthesis, cell proliferation, growth, migration, and apoptosis. The involvement of MAP kinase in noise-induced hearing loss (NIHL) has been implicated in the cochlea; however, it is unknown how expression levels of MAP kinase change after the onset of NIHL and whether they are regulated by transient phosphorylation or protein synthesis. CBA/J mice were exposed to 120-dB octave band noise for 2 h. Auditory brainstem response confirmed a component of temporary threshold shift within 0–24 h and significant permanent threshold shift at 14 days after noise exposure. Levels and localizations of phospho- and total- MEK1/ERK1/2/p90 RSK, JNK/c-Jun, and p38 MAPK were comprehensively analyzed by the Bio-Plex® Suspension Array System and immunohistochemistry at 0, 3, 6, 12, 24 and 48 h after noise exposure. The phospho-MEK1/ERK1/2/p90 RSK signaling pathway was activated in the spiral ligament and the sensory and supporting cells of the organ of Corti, with peaks at 3–6 h and independently of regulations of total-MEK1/ERK1/2/p90 RSK. The expression of phospho-JNK and p38 MAPK showed late upregulation in spiral neurons at 48 h, in addition to early upregulations with peaks at 3 h after noise trauma. Phospho-p38 MAPK activation was dependent on upregulation of total-p38 MAPK. At present, comprehensive data on MAP kinase expression provide significant insight into understanding the molecular mechanism of NIHL, and for developing therapeutic models for acute sensorineural hearing loss.

## Introduction

Noise-induced hearing loss (NIHL) is a major form of acute sensorineural hearing loss (SNHL). The audiological features and cochlear morphology of NIHL are well characterized, but the molecular process in the development of NIHL is not well-elucidated in the cochlea. In an animal study [1], exposure of mice to 120-dB octave band noise for 2 h resulted in immediate elevation of the auditory brainstem response (ABR) threshold and partial recovery of hearing at 24 h after the noise exposur*e.* The ABR threshold showed no significant change after 24 h, 3 days, 1 week, 2 weeks, and 8 weeks. Stabilization of a permanent threshold shift was confirmed at 2 weeks post-noise exposure.

Mitogen-activated protein kinases (MAP kinases) are serine/threonine-specific protein kinases that are activated by phosphorylation in response to a variety of extracellular stimuli. Conventional MAP kinases comprise three intracellular signaling pathways: MEK1 (mitogen-activated protein kinase kinase 1)/ERK 1/2 (extracellular signal-regulated kinases 1/2)/p90 RSK (p90 ribosomal S6 kinase), JNK (c-Jun amino (N)-terminal kinase)/c-Jun, and p38 MAPK pathways. These pathways coordinately regulate gene expression, mitosis, metabolism, motility, cell survival, apoptosis, and differentiation [2]. Sequential phosphorylation of MEK1/ERK1/2/p90 RSK is induced by growth factors, including platelet-derived growth factor, epidermal growth factor and nerve growth factor, cytokines, osmotic stress, and microtubule disorganization [3]. JNK/c-Jun phosphorylation is promoted by stress stimuli including heat shock, ionizing radiation, oxidative stress, DNA-damaging agents, cytokines, UV irradiation, and protein synthesis inhibitors [4]. The p38 MAPK pathway is also strongly activated by various environmental stresses, such as oxidative stress, hypoxia, ischemia, and UV irradiation [5].

The involvement of the MAP kinase pathways in NIHL is suggested by alteration in the cochlear expressions of phosphorylated MAP kinases after intense noise exposure, which causes a temporary or permanent threshold shift (TTS or PTS) in hearing [6]. Of these MAP kinases, JNK and p38 MAPK can function as stress-induced regulators of apoptosis. The protection of inner ear function by JNK inhibitor and p38 MAPK inhibitor after ischemic inner ear damage and NIHL have raised the possibility of using these inhibitors as therapeutic reagents for acute sensorineural hearing loss [7], [8], [9].

However, comprehensive analyses of MAPK expressions after noise trauma have not been conducted in the *in vivo* cochlea. Importantly, the precise time course of the regulation of MAP kinases during the development of NIHL is unknown. It is also unknown whether MAP kinases are regulated by transient phosphorylation or *de novo* synthesis of the proteins during this process.

The time course and the mode of regulation of MAP kinase proteins would be critical information to understanding the involvement of MAP kinases in NIHL and the development of therapeutic models for SNHL by interventions into MAP kinase pathways. In the present study, the amount of the phosphorylated proteins – phospho-MEK1, phospho-ERK1/2, phospho-p90 RSK, phospho-JNK, phospho-c-Jun, and phospho-P38 MAPK – as well as that of the total proteins – total-MEK1, total-ERK1/2, total-p90 RSK, total-c-Jun and total-P38 MAPK – were comprehensively analyzed at 0, 3, 6, 12, 24 and 48 h in the mouse cochlea after intense noise exposure. The changes in the hearing threshold showing TTS at 0, 12, and 24 h, and PTS at 14 days were confirmed after the noise trauma. Immunohistochemical data were also sought to delineate in which cochlear structure the active phosphorylated MAP kinases are expressed.

## Materials and Methods

### Animals

Male CBA/J mice at 10 weeks of age (Jackson Laboratory, Bar Harbor, ME, USA) without any evidence of middle ear infection were used for auditory brainstem response (ABR), protein extraction, and immunohistochemistry examination before and after intense noise exposure. All experimental protocols were approved by Okayama University's Committee on Use and Care of Animals (OKU-2011509) and in accordance with the recommendations of the Weatherall report, “The use of non-human primates in research”.

### Noise exposure

Animals were exposed, unanesthetized, and unrestrained in a cylindrical sound chamber (33 cm in diameter ×35 cm in height) with a speaker (CF1, Tucker Davis Technologies [TDT], Gainesville, FL, USA) on the top of the chamber. The stimulus of noise exposure was an octave band noise (8.0–16.0 kHz) presented at 120-dB sound pressure level (SPL) for 2 h. The exposure stimulus was generated and filtered with a 60 dB/octave slope by a sound generator (Rp 2.1, TDT), amplified (SA1, TDT), and delivered through the exponential speaker. Sound exposure levels were measured by a sound level meter and confirmed to be 120 dB across any position at the bottom of the cylindrical sound chamber.

### ABR threshold determination

One day before (control, *n* = 8) and at 0 h (immediately after the noise exposure, *n* = 8), 12 h (*n* = 7), 24 h (*n* = 7) and 14 days (*n* = 6) after the intense noise exposure, the animals were anesthetized by intraperitoneal injections of ketamine (80 mg/kg) and xylazine (8 mg/kg). ABRs were evoked with clicks through a sound conduction tube and recorded by needle electrodes inserted through the skin (vertex to the ipsilateral retroauricle with a ground at the contralateral retroauricle). The responses were recorded using a signal processor (RA16, TDT). Stimulus sounds were clicks (0.001 ms rise/fall time) with a plateau of 0.1 ms and stimulus rate of 21 Hz. The responses were processed through a 300- to 3,000-Hz bandpass filter and averaged 999 times. The stimuli were applied in 5-dB steps. ABR thresholds were defined as the lowest sound level at which the response peaks clearly presented were read by eye from stacked waveforms.

### Quantification of phosphorylated and total MAP kinases using the Bio-Plex® Suspension Array System

The Bio-Plex® Suspension Array System (Bio-Rad Laboratories, Hercules, CA, USA) enables simultaneous quantification of multiple phosphorylated and total proteins in each well of 96-well plates. In this system, an antibody directed against the desired target protein is covalently coupled to internally dyed beads with a fluorescence whose wavelength is specific for each target protein. The beads-coupled antibodies are allowed to react with lysate samples containing the target proteins. Then biotinylated detection antibodies specific for different epitopes of the proteins are added to the reaction, followed by an addition of streptavidin-phycoerythrin (streptavidin-PE). A dual-laser, flow-based microplate reader system (Bio-Plex® 200, Bio-Rad) detects the internal fluorescence of the individual dyed beads and the signal intensity on the bead surface. The relative abundance of the each target protein is reported as the ratio of fluorescence among the wells. In the present study, the abundance of phospho-MEK1, phospho-ERK1/2, phospho-p90 RSK, phospho-JNK, phospho-c-Jun, phospho-P38 MAPK, total-MEK1, total-ERK1/2, total-p90 RSK, total-c-Jun and total-P38 MAPK was simultaneously quantified in the cochlear samples. A specific beads-coupled antibody against mouse total-JNK is not available in the Bio-Plex® Suspension Array System.

Before (control, *n* = 8) and at 0 h (*n* = 8), 3 h (*n* = 8), 6 h (*n* = 8), 12 h (*n* = 8), 24 h (*n* = 8) and 48 h (*n* = 8) after intense noise exposure, the animals were deeply anesthetized by excess ketamine (200 mg/kg). The blood was removed by intracardiac perfusion of PBS and one cochlea per animal was promptly dissected and immediately frozen in liquid nitrogen until protein extraction. The sample tissue was homogenized in the lysis solution of the Bio-Plex® Cell Lysis kit (Bio-Rad) containing PMSF. The sample was sonicated and centrifuged at 4,500 g for 4 min. The supernatant was collected and the protein concentration was determined using the *DC* Protein Assay Kit II (Bio-Rad) and a spectrophotometer. Thirty to fifty mg of total protein was extracted per cochlea. One μL of each diluent containing beads-coupled antibodies against the multiple target proteins was mixed, diluted to a final volume of 50 μL in wash buffer, dispensed into 96-well plates (50 μL per well), and vacuum-filtered. Fifty μL of the protein samples from the cochleae, containing 27.7 μg of the total proteins, was dispensed into the wells, incubated overnight at room temperature, vacuum-filtered, and washed 3 times. Twenty-five μL of the biotinylated detection antibody diluent (25×) was then added, incubated for 30 min, vacuum-filtered and washed 3 times, followed by 50 μL of the streptavidin-PE diluent (100×), incubated for 10 min, and vacuum-filtered. After 3 rinses, 125 μL of the resuspension buffer was added and incubated for 30 min.

The plates were placed on the platform of the Bio-Plex® 200. Using Bio-Plex® Manager software (Bio-Rad), the wavelength of the fluorescence of the coupled beads, which is specific for the each target protein, was detected. The fluorescence from the streptavidin-PE was simultaneously detected and quantified for each target protein. The relative abundance of the target protein was calculated as [fluorescence intensity – background fluorescence] and expressed as the percentage to the mean of the control values from the cochleae that were not exposed to the noise. The data are presented as the means from more than triplicate detection of the proteins.

### Immunohistochemistry

After 3 h (for detection of phospho-p90 RSK and phospho-JNK) and 48 h (for detection of phospho-JNK and phospho-p38 MAPK) after the noise exposure, the animals were anesthetized and perfused intracardially, first with PBS for blood removal, then with 4% paraformaldehyde before the cochleae were dissected. After making small holes at the apex and the round window with a 27-gauge needle, the tissues were immersion-fixed at 4°C for 20 h. They were decalcified in 10% EDTA at 4°C for 4 days, dehydrated through graded alcohol and xylene, and embedded in paraffin. Paraffin sections of 6-μm thickness were dewaxed. Heat-induced epitope retrieval was performed by microwave for 3 min for the detection of phospho-JNK. The sections were incubated with rabbit polyclonal antibody against phospho-p90 RSK(Thr359/Ser363) (×1/250, 9344S, Cell Signaling Technology, Danvers, MA, USA), rabbit polyclonal antibody against phospho-JNK(Thr183/Tyr185) (×1/25, ab4821, Abcam, Cambridge, MA, USA) or rabbit monoclonal antibody against phospho-p38MAPK(Thr180/Tyr182) (×1/50, 4631S, Cell Signaling Technology), 10% normal goat serum, and 1% bovine serum albumin at 4°C overnight. Bound antibody was visualized by the ABC-DAB method (Vectastain Elite ABC kit, Vector Laboratories, Burlingame, CA, USA). Sections after reactions omitting the primary antibody served as the negative controls.

### Statistical analysis

For the data analyses of ABR thresholds and the Bio-Plex® Suspension Array System, the data were expressed by dB SPL and the percentages to the mean signal intensities of the control groups(animals without noise exposure), respectively. The differences among all the experimental groups(control, 0, 12, 24 h and 14 days for ABR threshold; control, 0, 3, 6, 12, 24 and 48 h for the Bio-Plex® Suspension Array System) were compared to each other non-parametrically by the Kruskal-Wallis test. Then the differences between the each group and the control group were tested by the Mann-Whitney *U*-test. Statistical significance was assigned to *p*-values of <0.01. Analyses were performed using SPSS software(IBM Corporation, Armonk, NY, USA).

## Results

### ABR thresholds

**Figure 1:**
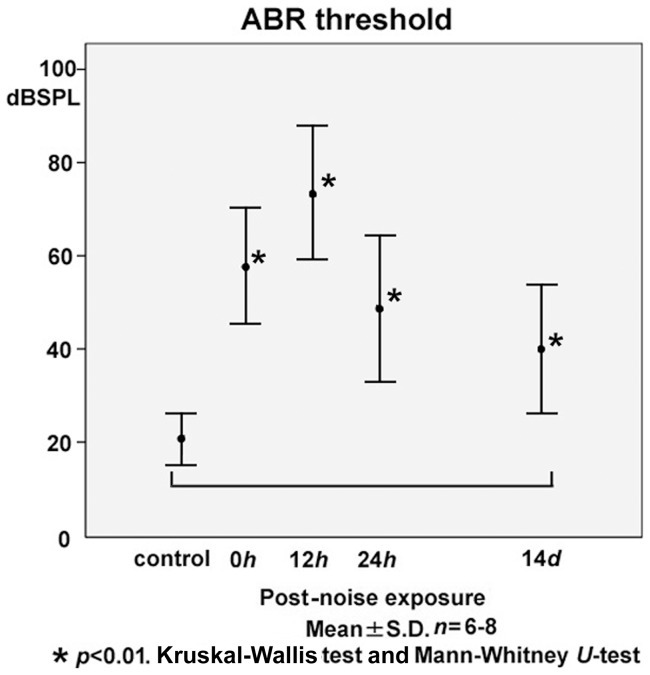
Time course of shifts in the auditory brainstem response (ABR) threshold following exposure to 120-dB octave band noise (8.0–16.0kHz) for 2 h. Significant elevation of the ABR threshold at 0 h (immediately after the noise exposure; 58.1±12.5 dB sound pressure level (SPL)), 12 h (73.6±14.4), and 24 h (48.6±15.7) exhibited a component of temporary threshold shift (TTS) during 0–24 h. The TTS was more severe at 12 h than at 0 h, and partial recovery was observed at 24 h. A significant permanent threshold shift was confirmed at 14 days (40.0±13.8), compared with the control level before the noise exposure (20.6±5.6 dB SPL).

ABR thresholds at 0 h (58.1±12.5 dB SPL), 12 h (73.6±14.4), 24 h (48.6±15.7) and 14 days (40.0±13.8) after the intense noise exposure were significantly elevated compared with the control level before the noise exposure (20.6±5.6 dB SPL) at all time points examined (Fig. 1). The temporary threshold shift (TTS) was more severe at 12 h than immediately after the noise exposure, and partial recovery was observed at 24 h. The component of TTS was observed during 0–24 h after the noise exposure. A significant permanent threshold shift (PTS) was confirmed at 14 days after the noise trauma.

### Quantification of phosphorylated and total MAP kinases using the Bio-Plex® Suspension Array System

In the present experiments, both phosphorylated and total MAP kinases were quantitatively examined, as phospho- MEK1, ERK1/2, p90 RSK, JNK, c-Jun, and p38 MAPK are the active forms of the each proteins, which further regulate the downstream signaling pathways including several transcription factors.

### MEK1

**Figure 2:**
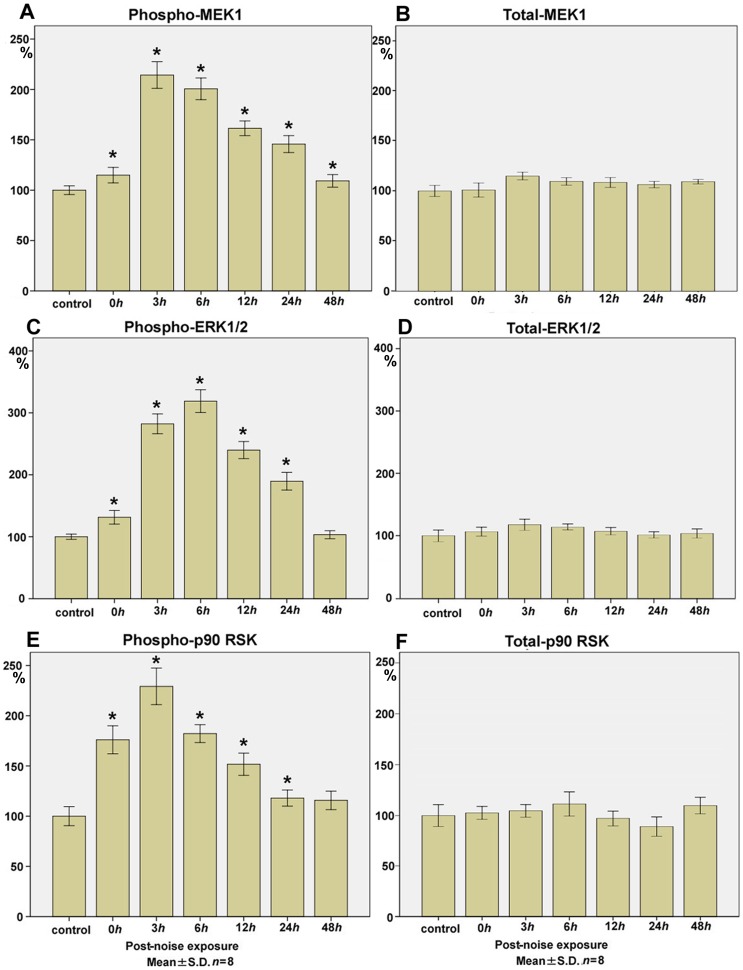
Time course of phospho- and total- MEK1, ERK1/2, p90 RSK expression in the cochlear lysate following exposure to the intense noise. The levels of phospho-MEK1 (**A**) at 0 h (immediately after the noise exposure;115.0±7.7; *p<*0.01), 3 h (214.3±13.3; *p*<0.01), 6 h (200.6±10.8; *p*<0.01), 12 h (161.5±7.3; *p*<0.01), 24 h (145.7±8.4; *p<*0.01), and 48 h (109.2±6.3; *p<*0.01) significantly increased to more than 2-fold after the noise exposure (control level; 100.0±4.3), with a peak surge at 3 h. Total-MEK1 levels (**B**) at 0 h (100.9±7.0), 3 h (114.8±3.8), 6 h (109.5±3.8), 12 h (108.4±4.9), 24 h (106.3±3.2), and 48 h (109.3±2.3) remained within 100±20% of the control level (100.0±5.5) over the time points. The levels of phospho-ERK1/2 (**C**) at 0 h (131.4±11.0; *p<*0.01), 3 h (282.3±16.0; *p*<0.01), 6 h (318.9±18.4; *p*<0.01), 12 h (239.9±13.8; *p<*0.01), 24 h (189.7±14.3; *p<*0.01), and 48 h (103.4±6.6) significantly increased to more than 3-fold after the noise exposure (control level; 100.0±4.3), with a peak surge at 6 h. Total-ERK 1/2 level (**D**) at 0 h (106.5±7.1), 3 h (117.8±9.1), 6 h (113.8±4.5), 12 h (107.0±5.8), 24 h (101.2±4.9), and 48 h (103.6±7.4) remained within 100±20% of the control level (100.0±9.6) at all time points examined. The levels of phospho-p90 RSK (**E**) at 0 h (176.1±14.0; *p<*0.01), 3 h (229.3±18.2; *p*<0.01), 6 h (182.2±8.9; *p*<0.01), 12 h (151.7±11.1; *p<*0.01), 24 h (118.0±8.0; *p<*0.01), and 48 h (115.8±9.2) significantly increased to more than 2-fold after the noise exposure (control level; 100.0±9.5), with a peak surge at 3 h. Total-p90 RSK levels (**F**) at 0 h (102.7±6.4), 3 h (104.8±6.5), 6 h (111.5±11.9), 12 h (97.2±7.6), 24 h (89.0±9.6) and 48 h (109.9±8.2) remained within 100±20% of the control level (100.0±10.9) without any surge over the time points. (*p<0.01, Kruskal-Wallis test and Mann-Whitney *U*-test; *n* = 8 for each time point).

The levels of phospho-MEK1 (Fig. 2A) at 0 h (immediately after the noise exposure; 115.0±7.7; mean±SD; *n* = 8; *p*<0.01, compared with the control level), 3 h (214.3±13.3; *p*<0.01), 6 h (200.6±10.8; *p*<0.01), 12 h (161.5±7.3; *p*<0.01), 24 h (145.7±8.4; *p*<0.01), and 48 h (109.2±6.3; *p<*0.01) after the noise exposure (control level; 100.0±4.3) was significantly upregulated to more than 2-fold with a peak surge at 3 h.

On the contrary to the surge in the levels of phospho-MEK1, total-MEK1 levels (Fig. 2B) at 0 h (100.9±7.0), 3 h (114.8±3.8), 6 h (109.5±3.8), 12 h (108.4±4.9), 24 h (106.3±3.2), and 48 h (109.3±2.3) after the noise exposure (control level; 100.0±5.5) remained within 100±20% over the time points.

### ERK1/2

The levels of phospho-ERK1/2 (Fig. 2C) at 0 h (131.4±11.0; *p<*0.01), 3 h (282.3±16.0; *p*<0.01), 6 h (318.9±18.4; *p*<0.01), 12 h (239.9±13.8; *p<*0.01), 24 h (189.7±14.3; *p<*0.01), and 48 h (103.4±6.6) after the noise exposure (control level; 100.0±4.2) showed more than a 3-fold increase, with a peak at 6 h, whereas total-ERK1/2 levels (Fig. 2D) at 0 h (106.5±7.1), 3 h (117.8±9.1), 6 h (113.8±4.5), 12 h (107.0±5.8), 24 h (101.2±4.9), and 48 h (103.6±7.4) after the noise exposure (control level; 100.0±9.6) were within 100±20% throughout the time points examined.

### p90 RSK

The levels of phospho-p90 RSK (Fig. 2E) at 0 h (176.1±14.0; *p<*0.01), 3 h (229.3±18.2; *p*<0.01), 6 h (182.2±8.9; *p*<0.01), 12 h (151.7±11.1; *p<*0.01), 24 h (118.0±8.0; *p<*0.01), and 48 h (115.8±9.2) after the noise exposure (control level; 100.0±9.5) significantly increased to more than 2-fold with a surge at 3 h.

Similarly to the levels of total-MEK1 and total-ERK 1/2, those of total-p90 RSK (Fig. 2F) at 0 h (102.7±6.4), 3 h (104.8±6.5), 6 h (111.5±11.9), 12 h (97.2±7.6), 24 h (89.0±9.6), and 48 h (109.9±8.2) after the noise exposure (control level; 100.0±10.9) remained within 100±20 without any surge throughout the time points.

### JNK

**Figure 3:**
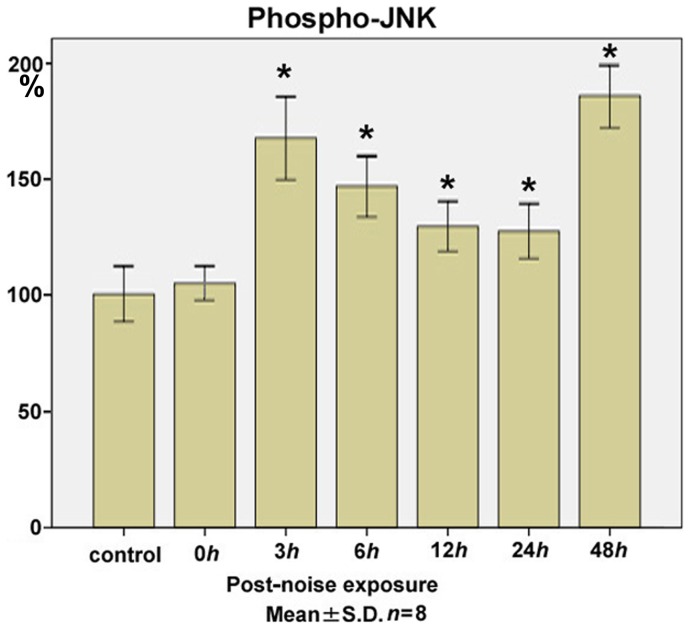
Time course of phospho-JNK expression in the cochlear lysate following exposure to the intense noise. The levels of phospho-JNK in the cochlear lysate at 0 h (immediately after the noise exposure; 104.7±7.6), 3 h (167.2±18.1; *p*<0.01), 6 h (146.4±13.3; *p*<0.01), 12 h (129.2±10.8; *p<*0.01), 24 h (127.1±11.8; *p<*0.01), and 48 h (185.4±13.8; *p<*0.01) after the noise exposure significantly increased from the control level (100.0±11.8), with the early and late peaks at 3 h and 48 h. (*p<0.01, Kruskal-Wallis test and Mann-Whitney *U*-test; *n* = 8 for each time point).

The levels of the phospho-JNK (Fig. 3) in the cochlear lysate at 0 h (104.7±7.6), 3 h (167.2±18.1; *p*<0.01), 6 h (146.4±13.3; *p*<0.01), 12 h (129.2±10.8; *p<*0.01), 24 h (127.1±11.8; *p<*0.01), and 48 h (185.4±13.8; *p<*0.01) after the noise exposure significantly increased from the control level (100.0±11.8), with the early and late peaks at 3 h and 48 h after the noise trauma.

### c-Jun

**Figure 4:**
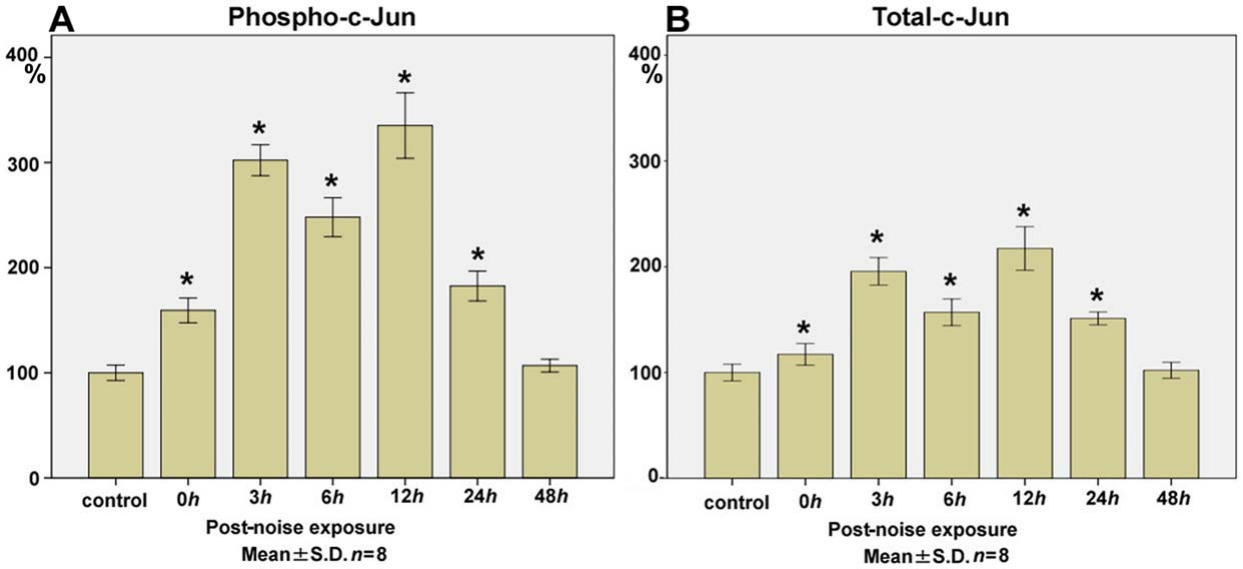
Time course of phospho- and total-c-Jun expression in the cochlear lysate following exposure to the intense noise. The levels of phospho-c-Jun (**A**) in the cochlear lysate at 0 h (immediately after the noise exposure; 159.3±11.8; *p<*0.01), 3 h (302.3±14.8; *p*<0.01), 6 h (248.0±18.5; *p*<0.01), 12 h (335.2±31.2; *p<*0.01), 24 h (182.6±14.2; *p<*0.01), and 48 h (106.9±6.1) after the noise exposure significantly increased from the control level (100.0±7.3) with the biphasic peak at 3 h and 12 h. Total-c-Jun levels (**B**) at 0 h (117.3±10.2; *p<*0.01), 3 h (195.7±13.0; *p*<0.01), 6 h (157.0±12.6; *p*<0.01), 12 h (217.4±20.6; *p<*0.01), 24 h (151.2±6.1; *p<*0.01), and 48 h (102.1±7.6) also showed significant increases from the control level (100.0±7.9) with the biphasic peak, which corresponds to the peaks in phospho-c-Jun at 3 h and 12 h. (*p<0.01, Kruskal-Wallis test and Mann-Whitney *U*-test; *n* = 8 for each time point).

The levels of phospho-c-Jun (Fig. 4A) at 0 h (159.3±11.8; *p<*0.01), 3 h (302.3±14.8; *p*<0.01), 6 h (248.0±18.5; *p*<0.01), 12 h (335.2±31.2; *p<*0.01), 24 h (182.6±14.2; *p<*0.01), and 48 h (106.9±6.1) after the noise exposure (control level; 100.0±7.3) significantly increased with a biphasic surge at 3 h and 12 h.

The levels of total-c-Jun (Fig. 4B) at 0 h (117.3±10.2; *p<*0.01), 3 h (195.7±13.0; *p*<0.01), 6 h (157.0±12.6; *p*<0.01), 12 h (217.4±20.6; *p<*0.01), 24 h (151.2±6.1; *p<*0.01), and 48 h (102.1±7.6) after the noise exposure (control level; 100.0±7.9) showed a significant corresponding biphasic surge to the changes in the levels of phospho-c-Jun.

### p38 MAPK

**Figure 5:**
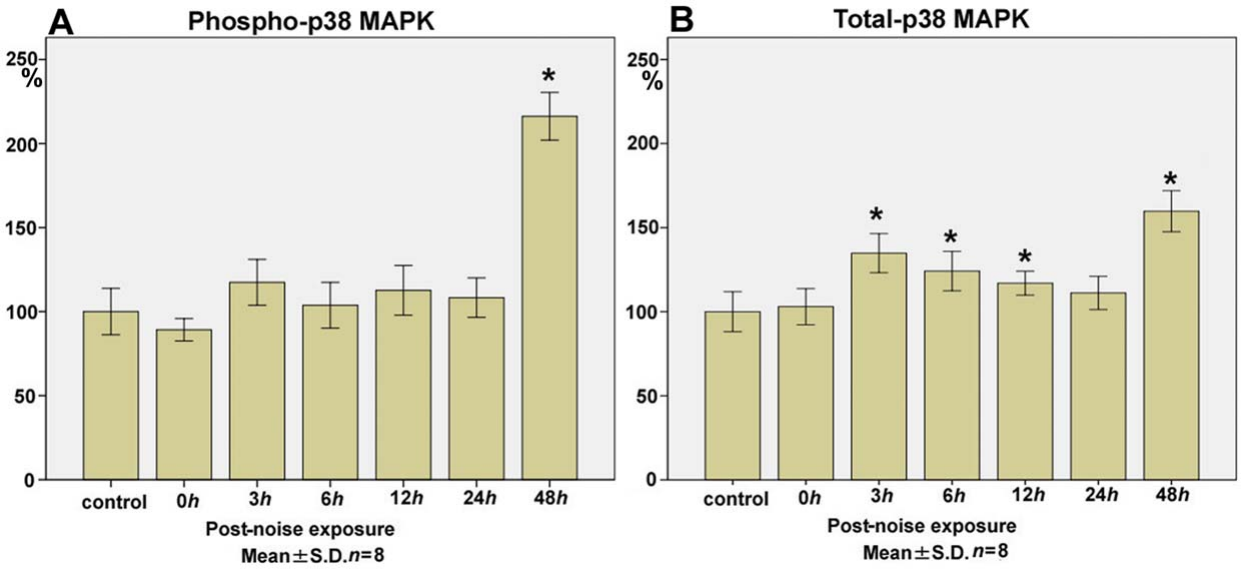
Time course of phospho- and total- p38 MAPK expression in the cochlear lysate following exposure to the intense noise. The levels of phospho-p38 MAPK (**A**) at 0 h (immediately after the noise exposure; 89.2±6.6), 3 h (117.4±13.6), 6 h (103.8±13.6), 12 h (112.6±14.8), 24 h (108.3±11.8), and 48 h (216.3±14.2; *p*<0.01) significantly increased to more than 2-fold of the control level (100.0±13.8) at 48 h after the noise exposure. Total-p38 MAPK levels (**B**) at 0 h (103.0±10.7), 3 h (134.8±11.6; *p*<0.01), 6 h (124.2±11.7; *p*<0.01), 12 h (116.9±7.1; *p*<0.01), 24 h (111.1±9.9), and 48 h (159.7±12.2; *p*<0.01) showed significant increases from the control level (100.0±11.9) at the late phase of 48 h, which coincided with the increase in phospho-p38 MAPK. The upregulation of total-p38 MAPK was also significant at 3 h, followed by 6 h and 12 h after the noise exposure. (*p<0.01, Kruskal-Wallis test and Mann-Whitney *U*-test; *n* = 8 for each time point).

The levels of phospho-p38 MAPK (Fig. 5A) at 0 h (89.2±6.6), 3 h (117.4±13.6), 6 h (103.8±13.6), 12 h (112.6±14.8), 24 h (108.3±11.8), and 48 h (216.3±14.2; *p*<0.01) after the noise exposure (control level; 100.0±13.8) showed a significant late surge at 48 h, to more than 2-fold of the control level.

Total-p38 MAPK levels (Fig. 5B) at 0 h (103.0±10.7), 3 h (134.8±11.6; *p*<0.01), 6 h (124.2±11.7; *p*<0.01), 12 h (116.9±7.1; *p*<0.01), 24 h (111.1±9.9), and 48 h (159.7±12.2; *p*<0.01) after the noise exposure (control level; 100.0±11.9) exhibited a significant surge at 48 h, which corresponds to the late increase in the phospho-p38 MAPK levels. The increase in the level of total-p38 MAPK was also significant at 3 h after the noise trauma.

### Immunohistochemistry

As the key markers for each pathway of the MEK1/ERK1/2/p90RSK, JNK/c-Jun, and p38 MAPK cascades, immunolocalizations of phospho-p90RSK, phospho-JNK and phospho-p38 MAPK were investigated in the cochlea. Immunohistochemical examinations were performed at the time points of the peaks in the expression of each phosphorylated protein (at 3 h and 48 h for phospho-JNK, 3 h for phospho-p90 RSK, and 48 h for phospho-p38 MAPK) in the cochlear lysate.

### Phospho-JNK

**Figure 6:**
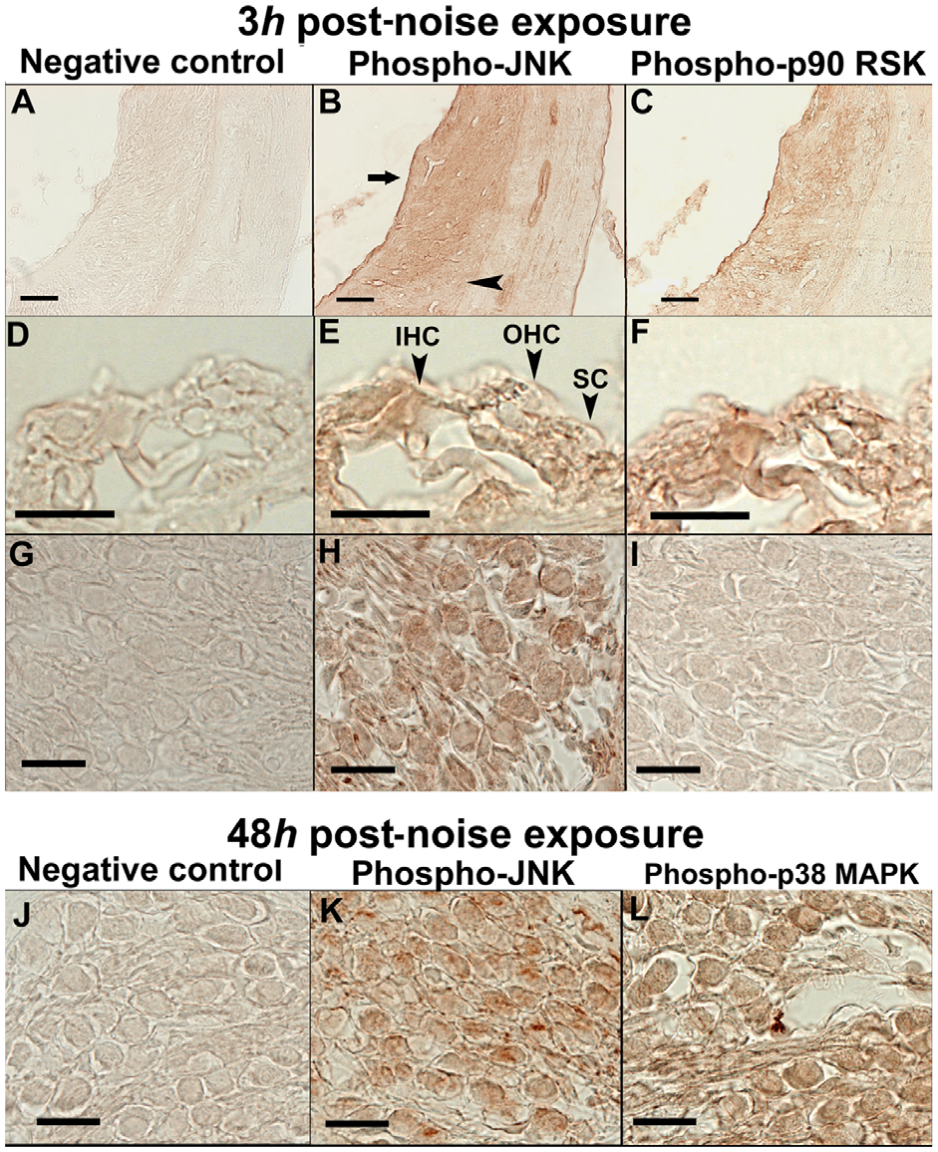
Immunolocalizations of phospho-JNK, phospho-p90 RSK, and phospho-p38 MAPK at 3 h and 48 h after the noise exposure. At 3 h post-noise exposure, cytoplasmic and nucleoplasmic immunoreactivity to phospho-JNK was observed in the spiral ligament (**B**), the sensory and supporting cells of the organ of Corti (**E**; OHC, outer hair cells; IHC, inner hair cells; SC, supporting cells) and the spiral neurons (**H**). In the spiral ligament, phospho-JNK immunoreactivity was more evident in the type I and II fibrocytes (**B, arrow**) than in the type III and IV fibrocytes (**B, arrowhead**). At 3 h post-noise exposure, immunoreactivity to phospho-p90 RSK was demonstrated in the spiral ligament (**C**) and in the sensory and supporting cells of the organ of Corti (**F**). Phospho-p90 RSK immunoreactivity was absent in the spiral neurons at this time point (**I**). At 48 h post-noise exposure, immunolocalization of phospho-JNK was shown in the cytoplasm and nucleoplasm of the spiral neurons (**K**). Phospho-p38 MAPK immunoreactivity in the cytoplasm and nucleoplasm was also demonstrated in the spiral neurons (**L**) at 48 h. The sections of negative controls resulted in no staining in any structures and time points (**A, D, G, J**). Scale bars indicate 20 (**D, E, F, G, H, I, J, K, L**) and 40 (**A, B, C**) μm.

At the early phase of 3 h post-noise exposure, nucleoplasmic and cytoplasmic immunoreactivity to phospho-JNK was observed in the spiral ligament (Fig. 6B), the sensory and supporting cells of the organ of Corti (Fig. 6E; OHC, outer hair cells; IHC, inner hair cells; SC, supporting cells) and the spiral neurons (Fig. 6H). In the spiral ligament, the immunolabeling was more evident in the type I and II fibrocytes (Fig. 6B, arrow) than in the type III and IV fibrocytes (Fig. 6B, arrowhead).

At the late phase of 48 h post-noise trauma, unequivocal immunoreactivity to phospho-JNK was observed in the spiral neurons (Fig. 6K).

### Phospho-p90 RSK

At 3 h post-noise trauma, when phospho-p90 RSK expression reached the maximum in the cochlear lysate, immunoreactivity to phospho-p90 RSK was observed in the spiral ligament (Fig. 6C) and in the sensory and supporting cells of the organ of Corti (Fig. 6F). In contrast to phospho-JNK at this time point, no immunolabeling for phospho-p90 RSK was observed in the spiral neuron (Fig. 6I).

### Phospho-p38 MAPK

At 48 h post-noise exposure, immunoreactivity to phospho-p38 MAPK was demonstrated in the nucleoplasm and cytoplasm of the spiral neurons (Fig. 6L).

In all experiments to detect the phospho-MAP kinases, no significant signal was observed in the control sections (Fig. 6A, D, G, J).

## Discussion

**Figure 7:**
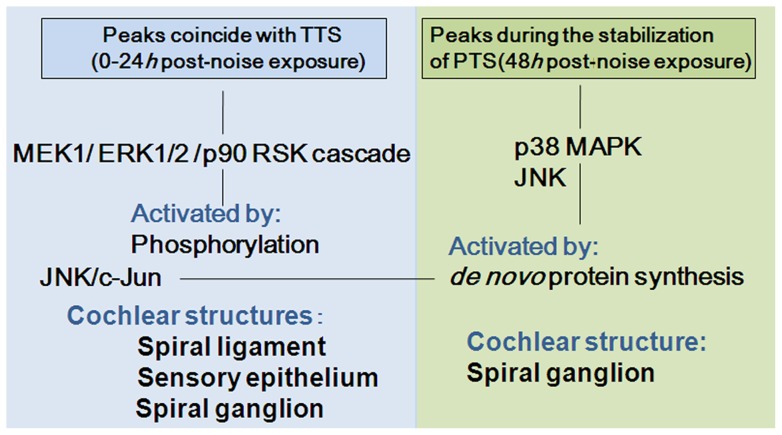
Summary of upregulation of MEK1/ERK1/2/p90 RSK, JNK/c-Jun and p38 MAPK in the cochlea after exposure to the intense noise. The phospho-MEK1/ERK1/2/p90 RSK signaling pathway was activated in the spiral ligament and in the sensory and supporting cells of the organ of Corti, with peaks at 3–6 h and independently of *de novo* synthesis of the protein kinases. The expression of phospho-JNK and p38 MAPK showed late upregulation in the spiral neurons at 48 h, in addition to early upregulation with peaks at 3 h after the noise trauma. Phospho-p38 MAPK and c-Jun activation was dependent on *de novo* synthesis of the proteins.

As summarized in Fig. 7, the comprehensive, collated data of MAP kinase expression delineated upregulation of the phospho-MEK1/phospho-ERK1/2/phospho-90 RSK cascade within the early phase of 0–24 h after the noise exposure, which coincided with a TTS. This process did not involve the temporal surge in the levels of total-MEK1, total-ERK1/2 and total-p90 RSK; hence, the upregulation of phospho-MEK1/phospho-ERK1/2/phospho-90 RSK was largely due to transient phosphorylation of the proteins and did not involve *de novo* synthesis of the proteins. Immunohistochemical data showed that the expression of phospho-p90 RSK occurred in the lateral wall (spiral ligament) and in the sensory and supporting cells of the cochlea at 3 h post-noise exposure, at the time of the peak surge of phospho-p90 RSK.

Phospho-JNK and phospho c-Jun, as well as total c-Jun, also showed surges beginning as early as 3 h after the noise exposure. The localization of phospho-JNK was demonstrated in the lateral wall (the spiral ligament), in the sensory and supporting cells of the organ of Corti, and in the spiral neurons at 3 h. The upregulation of a downstream effector, phospho-c-Jun, was dependent on the *de novo* synthesis of c-Jun.

In contrast to the upregulation of the phospho-MEK1/phospho-ERK1/2/phospho-90 RSK cascade within 0–24 h, the levels of phospho-JNK and phospho-p38 MAPK also demonstrated significant increases at the late phase of 48 h post-noise exposure. Total-p38 MAPK showed a significant and corresponding increase at 48 h; therefore, the upregulation of phospho-p38 MAPK at 48 h involved *de novo s*ynthesis of the protein. Immunohistochemical results showed that the expression of phospho-JNK and phospho-p38 MAPK occurred in the spiral neurons of the cochlea at this time point. ABR threshold testing indicated partial recovery of the TTS during the preceding 12–24 h and significant PTS at 14 days post-noise exposure.

Upstream from the MEK1/ERK1/2/p90 RSK signaling pathway, growth factor binding to receptor protein tyrosine kinases in the cell membrane triggers activation of a G-protein, Ras, by exchange of its guanosine diphosphate (GDP) to guanosine triphosphate (GTP) [10]. Activated Ras phosphorylates Raf, and in turn, activated Raf phosphorylates MEK1, leading to the sequential phosphorylation of ERK 1/2 and p90 RSK [11]. p90 RSK is a major downstream effector of the MEK1/ERK1/2 signaling pathway and mediates biological processes such as cell survival, protein synthesis, cell-proliferation, cell growth, and migration through the regulation of transcription factors, c-Fos, CREB (cAMP response element binding protein) and NF-kappa B (nuclear factor-kappa B) [12]. p90 RSK activates CREB kinase, which in turn phosphorylates and activates CREB. CREB initiates transcription of survival-promoting genes, including Bcl-2 (B-cell lymphoma 2), Bcl-xL (B-cell lymphoma-extra large) and Mcl1 (myeloid cell leukemia sequence 1), and promotes survival of cultured primary neurons *in vitro* [13], [14]. The physiological roles of this signaling pathway suggest that the upregulation of phospho-MEK1/phospho-ERK 1/2/phospho-p90 RSK with the peak surges during 3–6 h, as revealed in the present data, is a protective response to the noise trauma involving the sensory epithelium and the spiral ligament of the cochlea.

Activation of JNK by the upstream kinases MKK4 (MAP kinase kinase 4)/MKK7 [2] can be induced by acoustic trauma [15], ototoxic drugs, and electrode insertion [16] to the inner ear. Phosphorylated JNK binds and phosphorylates downstream effectors such as a transcription factor, c-Jun, ATF2 (activating transcription factor 2), Elk1 (E-twenty six-like transcription factor 1) and p53 (tumor protein 53) [17]. It is reported that these effectors mediate apoptosis in the sensory epithelium and in the lateral wall of the cochlea and neurons [7], [15], [18]. In the present experiments, phospho-JNK was upregulated in the sensory epithelium, in the lateral wall (the spiral ligament), and in the spiral neurons as early as 3 h post-noise trauma, which is consistent with the previous reports suggesting that the peak expression of phospho-JNK occurred at 0–12 h after the noise exposure that can induce PTS [7], [15]. The present data also provides a novel finding of the second, late surge of phospho-JNK in the spiral neurons at 48 h post-noise trauma.

AM111 peptide (which is an equivalent term to D-JNKI-1 peptide) is a cell-permeable compound that inhibits phospho-JNK activity. Intratympanically applied AM111 onto the round window protects hearing from acoustic trauma and prevents ischemic damage to the cochlea [7], [8], [19]. AM111 is currently under investigation as a potential therapeutic reagent to rescue acute sensorineural hearing loss (http://www.aurismedical.com/, 2013). The present data of the time course of phospho-JNK expression provides significant insight into the design of appropriate therapeutic protocols using the JNK inhibitor.

Phosphorylation of p38 MAPK by the upstream regulators MMK3/MMK6 [2] can be induced by acoustic trauma and aminoglycoside to the cochlea [9], [20], [21]. Activated p38 MAPK phosphorylates a number of substrates such as MSK1(mitogen- and stress-activated protein kinase 1)/MSK2 and MNK1(MAP kinase interacting serine/threonine kinase 1) [2]. The p38 MAPK signaling pathway shares the downstream effectors, including ATF2 and Elk1, with the JNK pathway and is also involved in apoptosis [17].

The p38 MAPK inhibitors SB202190 and SB203580 dose-dependently decreased hair cell loss and protected hearing after acoustic overexposure of the mouse cochlea [9]. In previous animal studies, the expression of phospho-p38 MAPK was observed in the sensory epithelium at 2–4 h post-noise exposure and the p38 MAPK inhibitors were injected into the mice immediately before the noise exposure [9], [21]. These studies have not addressed the cochlear expression of phospho-p38 MAPK for a longer time period than 4 h after noise exposure. The present data for the first time demonstrated the late upregulation of phospho-p38 MAPK in the spiral neurons at 48 h, which is dependent on *de novo* synthesis of the p38 MAPK protein. The phospho-p38MAPK level showed a tendency to increase at 3 h, but was not significant at this time point. We assume that in the present experiments, the sensitivity for detecting phospho-p38 MAPK did not reach the level of significance to show its upregulation in the cochlea at 3 h after noise exposure. The level of total-p38 MAPK demonstrated significant, early upregulation with a peak at 3 h, followed by 6 h and 12 h, which is consistent with previous reports [9], [21] and suggestive of the *de novo* protein synthesis.

The present data demonstrated activation of the MEK1/ERK1/2/p90 RSK signaling pathway in the spiral ligament and in the sensory and supporting cells of the organ of Corti, with the peaks occurring at 3–6 h and coinciding with the observed TTS after noise exposure. This process is independent of *de novo* protein synthesis and thought to be a protective response to noise trauma. It is generally accepted that JNK and p38 MAPK act as stress-induced kinases involved in apoptosis. In addition to the early upregulation, with the peak at 3 h after the noise exposure, the present data demonstrated the late upregulations of JNK and p38 MAPK pathways in the spiral neurons at 48 h after the noise trauma. The p38 MAPK activation is dependent on *de novo* protein synthesis. The comprehensive analysis of MAP kinase expression will be critical to understanding the molecular mechanism of NIHL and for developing therapeutic models for acute SNHL.
